# Dynamic Interplay between Social Brain Development and Nutrient Intake in Young Children

**DOI:** 10.3390/nu15173754

**Published:** 2023-08-28

**Authors:** Alexandros K. Kanellopoulos, Sarah Costello, Fabio Mainardi, Kyoko Koshibu, Sean Deoni, Nora Schneider

**Affiliations:** 1 Brain Health Department, Nestlé Institute of Health Sciences, Société des Produits Nestlé SA, Vers-Chez-les-Blanc, 1000 Lausanne, Switzerland; 2 Data Science Group, Nestlé Institute of Health Sciences, Société des Produits Nestlé SA, Vers-Chez-les-Blanc, 1000 Lausanne, Switzerland; 3 Advanced Baby Imaging Lab, Rhode Island Hospital, 1 Hoppin Street, Providence, RI 20903, USA; 4 Department of Radiology, Warren Alpert Medical School of Brown University, 222 Richmond St., Providence, RI 02912, USA; 5 Spinn Neuroscience, Seattle, WA 98275, USA

**Keywords:** nutrition, social brain, myelination, MRI, toddlers

## Abstract

Myelination of the brain structures underlying social behavior in humans is a dynamic process that parallels the emergence of social–emotional development and social skills in early life. Of the many genetic and environmental factors regulating the myelination processes, nutrition is considered as a critical and modifiable early-life factor for establishing healthy social brain networks. However, the impact of nutrition on the longitudinal development of social brain myelination remains to be fully understood. This study examined the interplay between childhood nutrient intake and social brain development across the first 5 years of life. Myelin-sensitive neuroimaging and food-intake data were analyzed in 293 children, 0.5 to 5 years of age, and explored for dynamic patterns of nutrient—social brain myelin associations. We found three data-driven age windows with specific nutrient correlation patterns, 63 individual nutrient–myelin correlations, and six nutrient combinations with a statistically significant predictive value for social brain myelination. These results provide novel insights into the impact of specific nutrient intakes on early brain development, in particular social brain regions, and suggest a critical age-sensitive opportunity to impact these brain regions for potential longer-term improvements in socio-emotional development and related executive-function and critical-thinking skills.

## 1. Introduction

Social skills refer to a wide range of abilities allowing individuals to interact and communicate effectively [1]. Social brain areas undergo significant maturation during infancy and toddlerhood, which lays the foundation for later social–emotional competence [2]. Thus, the development of social brain areas is crucial for an individual’s social skills and emotional development to positively cope with daily life, to build resilience and to integrate well in a society. Therefore, the early life development of social–emotional skills lays an essential foundation for later academic and social success [3], with evidence showing that social–emotional skills are associated with complex cognitive skills [4], early school transition [5,6], academic achievement [7,8], creative thinking [9], and the development of lasting friendships, social bonds, and positive well-being [10].

Myelination is a key process in neurodevelopment and can serve as a valuable biomarker of the brain’s functional maturity [11,12,13]. Though myelination continues into and throughout adulthood, it is most rapid and dynamic over the first 2–5 years of life [14], and myelination of specific brain regions and networks strongly overlaps with the emergence of specific cognitive functions [12,15,16]. Neuroimaging data further show that region-specific patterns and differential trajectories of myelin development across the first 5 years of life are related to cognitive development and ability [13,17,18,19]. These findings have recently been extended to social–emotional skills, with evidence suggesting that myelination of the social brain is significantly correlated with changes in social–emotional development in infants and toddlers, such as self-regulation, compliance, communication, adaptive functioning, autonomy, affect, and interaction with people, in the first 3 years of life [2,20]. This description of the neural basis of human social behavior, a network of connected regions that include the superior temporal sulcus (STS), anterior cingulate cortex (ACC), medial prefrontal cortex (mPFC), temporoparietal junction (TPJ), inferior frontal gyrus (IFG) and the anterior insula and amygdala [2], and its link with social–emotional skills or other cognitive functions in infants and toddlers, represents a potential opportunity to identify nutritional interventions to target social–emotional development specifically. However, only a few studies have investigated the longitudinal impact of nutrition on brain development considering these dynamics. This is particularly insightful, given the recent evidence showing myelin imaging to be a reliable marker for the assessment of the longitudinal nutritional impact on brain development in young children [21] and modifications to the brain’s structural maturation [11,22,23].

The non-homogeneous spatial and temporal development of the brain supports the notion that certain regions of the brain may be susceptible to specific nutrients [21] at certain points in time during development [24,25]. This forms the basis of one of the major principles of how nutrition may regulate brain development, and emphasizes the role of timing, dose, and duration of environmental influences such as nutrient exposure [25,26,27]. For example, some nutrients may have a broad or “global” effect (e.g., protein, energy) on the developing brain as a whole. In contrast, others, like micronutrients (e.g., iron) may have a more specific or regional impact [24]. With this understanding, the nutritional demands of the developing brain are suggested to be concomitant with the specific age and stage of brain development [24,25]. Therefore, we hypothesized that nutrients from food intake data would show differential association patterns with myelin development that are dependent on the age of the children, brain developmental stages, and brain areas.

Understanding the interplay between early-life nutrition and its effect on the social brain’s developmental dynamics is critical for identifying and investigating age- and brain stage-appropriate nutritional opportunities. However, there is limited research on brain–nutrient dynamics by age in healthy toddlers and preschool children. For example, from one to five years of age, observational and intervention studies have mainly investigated the influence of nutrition on social–emotional skills in populations with neurodevelopmental disorders such as autism that emphasize altered social skills as core symptoms [28,29,30,31]. To our knowledge, no study has explored the effect of nutrient intake on social brain maturation in a neurotypically-developing cohort.

Therefore, this study aimed to investigate stage-dependent changes in myelin associations related to age and the brain areas, specifically within the social brain regions in healthy and typically developing children from birth through 5 years of age. Previously, we demonstrated for the first time the impact of an enriched formulation on developmental myelination in term-born infants [32]. However, potential interactions and/or synergistic effects between nutrients on the developing brain of well-nourished infants and toddlers require further elucidation. Therefore, we extended our current study to identify nutrient combinations with a significant predictive value for social brain myelination, which may be foundational for later social–emotional outcomes.

## 2. Materials and Methods

### 2.1. Participants

Participants in this study were drawn from the RESONANCE Study of Prenatal and Postnatal Child Health and Development—a longitudinal birth cohort that is part of the Environmental influences on Child Health Outcomes (ECHO, echochildren.org) Program of the NIH. From the larger cohort of ~1700 mother-child dyads, 293 children between 6 months and 5 years were selected who met the following specific criteria listed in Supplementary Table S1. This study was approved by an institutional review board (IRB number 1828261). The demographic information for the selected study population is summarized in Table 1.

### 2.2. Myelin Magnetic Resonance Imaging (MRI) Data Acquisition and Processing

All MR imaging was performed on a 3 Tesla Siemens Tim Trio scanner during natural and unsedated sleep. Noise-derated imaging sequences, a sound-insulating MRI bore liner, and noise-reducing hearing protectors were used to minimize acoustic noise and facilitate sleep [33], and foam cushions and vacuum immobilizers were used to help limit child motion during the scan. For the oldest children who were able to tolerate scanning awake, a favorite movie or TV show was played to keep their attention and help limit motion. Age-optimized imaging protocols [14] were used to collect high-quality multi-component relaxometry data, which were used to calculate voxel wise measures of the myelin water fraction (MWF)—a surrogate measure of myelin content.

MWF processing followed previously described methods [21] that include removal of non-brain signal, linear alignment of the multicomponent data, calibration of B_0_ and B_1_ field inhomogeneities, and fitting of a three-pool tissue model to quantify the MWF. Each child’s MWF image was then non-linearly aligned to a custom study template for population analyses.

**Figure 1:**
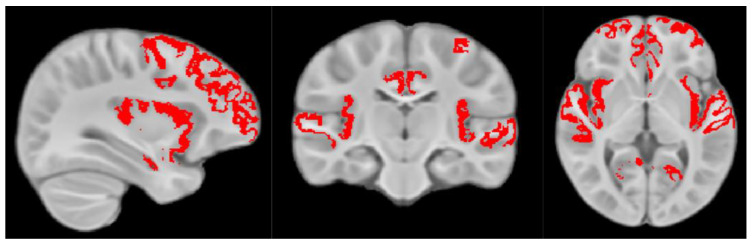
Overlay of the social brain mask on the study template. Brain region masks corresponding to superior temporal sulcus (STS), anterior cingulate cortex (ACC), medial prefrontal cortex (mPFC), temporoparietal junction (TPJ), inferior frontal gyrus (IFG) and the anterior insula and amygdala.

Using an anatomical atlas aligned to our study template, brain region masks corresponding to the social brain, including superior temporal sulcus (STS), anterior cingulate cortex (ACC), medial prefrontal cortex (mPFC), temporoparietal junction (TPJ), inferior frontal gyrus (IFG) and the anterior insula and amygdala (Figure 1) were superimposed on each child’s aligned data, and mean MWF values calculated.

### 2.3. Nutritional Data Collection

Nutrient intake data were also collected alongside and within 1 week of the neuroimaging data using the Automated Self-Administered 24-h dietary assessment tool (ASA-24) System [34]. The ASA-24 was filed by the parents, providing an average nutrient intake per day per child. Values for amino acids and phospholipids, including sphingomyelin, were retrieved from the relevant United States Department of Agriculture (USDA) databases [35]. The USDA choline database was used to provide values of free choline, glycerophosphocholine, phosphocholine, phosphatidylcholine and sphingomyelin from ~630 common foods. Because the USDA database does not encompass the full range of dietary variations across different populations and could introduce inaccuracies into the nutrient intake calculations, the following nutrient values were calculated from previous studies. Ganglioside values for meat and fish were estimated by Khor and his colleagues [36], and for milk both human and bovine were assumed to be 11 mg of ganglioside per liter, as suggested by Vesper et al. 1999 [37]. The oligosaccharides 3′SL and 6′SL are only found in bovine milk products; values of 114 and 23 mg/100 g were used for their respective concentrations in cow’s milk [38]. For the estimation of oligofructose, we based our calculations on Moshfegh et al. 1999 [39]. We acknowledge that these assumptions might not accurately reflect the actual nutrient content in the participants’ diets, which could lead to potential inaccuracies in the results.

We then mapped the foods reported in ASA-24 to the corresponding items in the USDA database and estimated the daily total nutrient intake. It is important to note that the ASA24 system includes a comprehensive database of over 17,000 portion-size image files linked to portions in the USDA’s Food and Nutrition Database for Dietary Studies (FNDDS). These images are freely available for use, and they play a crucial role in helping individuals estimate accurate portion sizes when completing the 24 h dietary assessment.

### 2.4. Dynamics of Social Brain Myelin–Nutrient Intake Associations

From the combined data from all 293 children, we used a sliding-window approach to examine the temporal dynamics of MWF-nutrient correlations, as described previously [21]. Briefly, we used a sliding window of 25 MWF measurements (72–162 days apart) and a general linear model to examine how nutrient intake affected regional MWF in children:

MWF = β_0_ + β_1_log(age) + β_2_Nutrient + β_3_Nutrient × log(age).

We plotted the *p*-values (unadjusted *p* < 0.05) of the nutrient term (β_2_) for each age-nutrient-brain myelin combination. We applied a Sobel filter edge detection to identify age windows with different nutrient associations. Nutrients per window were detected based on 50% significance for positive associations.

### 2.5. Nutrient Intake Dynamics per Nutrient–Social Brain Myelin Age Window

Previously, we identified three nutrient–myelin windows that cover the age range of 1–5 years, and these windows are aligned with reported myelin and white matter dynamics that change in the first 5 years [21]. The growth of myelin and white matter changes from fast and steep in window 1 to continued but slower growth in window 3. Window 2 possibly represents the inflection period [21]. Therefore, we provided evidence of the effect of nutrition on myelination involving positive nutrient correlations during the nutrient–myelin windows for the whole brain [21]. Here, we follow the same approach, specifically for the social brain regions. Using an edge-detection filter applied to the map of *p*-values for each nutrient × age combination, we identified the following age windows that appeared to show differing temporal profiles: 6–20 months (N = 88), 21–30 months (N = 48), and 31–60 months (N = 157). Within each of these age windows, and for each nutrient, the following linear model was fit to identify nutrients whose intake was positively or negatively associated with MWF. The linear model was described previously [21]. An increasing trend was defined as a positive and a decreasing trend as a negative slope in the linear model, with a *p*-value below 0.05. A non-significant slope was considered as a “stable trend”.

### 2.6. Identification of Window-Specific Nutrient Blends

The associations of myelination with combinations of nutrients were investigated. To that end, we applied regression models. Within each age window, the dataset containing the nutrient intakes, the age and the myelin water fraction was randomly split into a training dataset (75%) and a test dataset (25%). The training set was used to fit an elastic net [40] and a random forest [41], using a 10-fold cross-validation process. In each window, only the nutrients pre-selected as significantly associated with myelin (based on the sliding-window approach defined above) were used in the model. An elastic net is a penalized regression model, apt to select the most important predictors in a high-dimensional setting while being robust to collinearity among the predictors (indeed, high collinearity frequently occurs between the intakes of some nutrients belonging to the same category, for instance, fatty acids or amino acids). Random forests [41] implement an alternative regression approach, not relying on linearity assumptions. The fitted model was then used to predict the myelin water fraction values on the test set. The predicted values were then compared to the original values, through their Spearman correlation and the root-mean-squared error (RMSE).

In addition, we applied the random forest regression across the age windows and used a feature selection algorithm [42] to identify the nutrients that jointly predict MW across ages. From the original list of 88 nutrients, we removed redundancies (e.g., total phospholipids and total fats), non-essential amino acids, and nutrients for which there were safety concerns (methionine and phenylalanine) for children. This resulted in a list of 63 nutrients. The association between social brain myelin and nutrients was first investigated on the whole set of 63 nutrients (untargeted approach). Because our analysis is limited to one dietary assessment, we cannot exclude the possibility that other nutrients or another dietary assessment could reveal more nutrient–myelin associations.

The regression modelling with variable selection was then repeated for targeted nutrient categories: micronutrients (minerals and vitamins), lipids only, amino acids only, and fatty acids only. The reason for this targeted approach is because previous clinical and preclinical studies provide evidence for the role of these nutrients in the brain development and function and also in myelination. Macronutrients were not considered in these analyses, as they represent broad categories, and the aim was to investigate myelin associations with combinations of specific nutrients. Potential confounders, such as gender, socio-economic status, and maternal education were not included in any of the models, as gender and maternal education were not found to be significantly associated with MWF [21].

## 3. Results

### 3.1. Dynamic Nutrient Intake—Social Brain Window Associations

**Figure 2:**
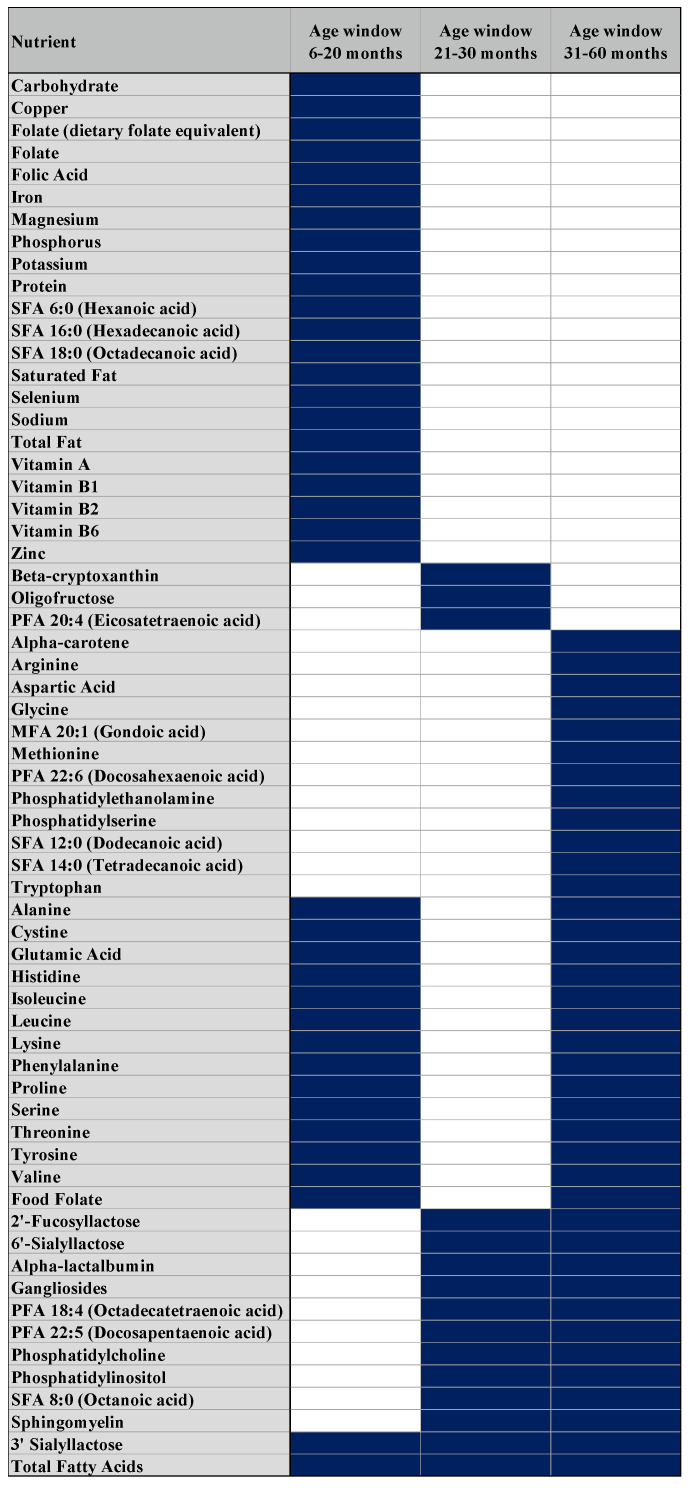
Nutrients significantly associated with myelin water fraction (MWF) (in dark blue) for each age window (6–20 months, 21–30 months, and 31–60 months).

From our sliding-window approach, three age windows: 6–20 months (N = 88), 21–30 months (N = 48), and 31–60 months (N = 157) were identified. Within these age windows, 28 nutrients were identified between 6 and 20 months of age as having a significant (positive or negative) association with MWF in the social brain. In the 21–30-month window, we identified 14 significant nutrients, and 27 in the 31–60-month window (Figure 2). Total fatty acids and 3′SL were common to all windows, while several phospholipids were overlapping between the second and third windows. Minerals were only significantly associated with MWF in the first age window.

### 3.2. Nutrient Intake across Age Windows

Furthermore, a myelin-independent correlation of individual nutrient intake and age windows showed different variation patterns and dynamics per each age window. The value ranges of nutrient intake were determined through the ASA-24 system. The average (mean), minimum and maximum (min-max) nutrient intake values of all the nutrients for each window are shown in Table 2.

### 3.3. Age Window-Specific Nutrient Blends Associated with Social Brain Myelination

Nutrient intakes are not independent of each other and can be highly correlated. To account for the non-linear associations and correlations between nutrient combinations, a regression model using a random forest algorithm to predict the social brain MWF from the nutrient intakes and the age was used. First, the variables selected across ages in the untargeted model were, in order of importance: Age, Gangliosides, Phosphatidylinositol, Sphingomyelin, Phosphorus, 3-SL, DHA, 6-SL, Phosphatidylserine, Linoleic acid, Calcium, ARA, Folate, Vitamin B12, α-Linolenic acid, and Beta-carotene. The predicted myelin association was highly correlated with the true values (r = 0.67).

The list of nutrients selected from the untargeted regression analysis and the Spearman correlation between predicted and actual values of MWF is reported in Table 3. The combination of nutrients for age window 6 to 20 months included B vitamins, minerals, and amino acids, while the combination for across ages primarily included HMOs and lipids. No specific nutrient combinations were identified for the other two age windows.

Lastly, a targeted analysis showed that combinations of amino acids, fatty acids and lipids alone significantly correlate with myelin across ages. These nutrient combinations showed a weaker association with myelin than the micronutrient combination (minerals and vitamins), which showed a comparable association in the untargeted approach (Table 4). No age-window-specific nutrient combinations were identified in the targeted analysis.

## 4. Discussion

Neuroimaging data suggest that region-specific patterns and distinct myelin developmental trajectories are associated with cognitive development and ability during the first five years of life. These findings have recently been extended to social–emotional skills in infants and young children. The uneven spatial and temporal development of the brain supports the hypothesis that certain regions of the brain may receive certain nutrients at certain times during development [21]. With this understanding, it is believed that the nutritional needs of the developing brain correlate with specific ages and stages of brain development. However, given these dynamics, few studies have examined the longitudinal effects of diet on brain development. This study extends our findings of dynamic nutrient intake-myelin development interplay in early life and is the first to examine that interplay for more specific domains of development, i.e., social brain myelination in healthy and well-nourished children. Furthermore, the study builds on previous work that found general brain–nutrient dynamics [21] and demonstrates the possibility of identifying age-adapted nutrient candidates for specific brain areas that underly specific developmental functions, such as social–emotional development [2].

Social brain myelination has previously been shown to influence the development of social–emotional skills in infants younger than 3 years of age [2]. Using myelin water fraction within the social brain regions as a marker for social brain maturation, this study revealed specific nutrients and combinations of nutrients (blends) that are important for supporting the myelin development of the social brain regions. Therefore, the social brain blends found in this study may support children’s different cognitive functions, including social–emotional skills, attention, language, and creative thinking, among others.

From 6 to 60 months, 16 nutrients were significantly associated with myelin, including polar lipids (gangliosides, phosphatidylinositol, sphingomyelin, phosphatidylserine, and phosphatidylcholine), minerals and vitamins (phosphorus, calcium, folate, vitamin B12), fatty acids (DHA, Linoleic acid, ARA, α-Linolenic acid), HMOs (3′-SL, 6′-SL) and β-carotene, with minerals and vitamins having stronger associations compared to other nutrient categories (Figure 2). Amino acids were the only nutrient category associated with myelin development during the first and third window of age, suggesting a differential role for these nutrients during the brain network connectivity process.

Previous clinical and preclinical research has provided a plethora of supporting evidence for the role of identified nutrients in brain development. The crucial importance of dietary lipids like DHA, ARA and sphingomyelin on brain development has been studied extensively [23,25,37,43,44,45,46,47,48]. Polar lipids (e.g., gangliosides, phosphatidylinositol, sphingomyelin, phosphatidylserine, and phosphatidylcholine) are structural components of neural tissues, with the peak rate of accretion overlapping with neurodevelopmental milestones [46,48]. The role of polar lipids in cognitive and social–emotional development is thought to be mediated through the regulation of signal transduction, myelination, and synaptic plasticity [48]. Preclinical data show that dietary sphingomyelin contributes to myelination of the central nervous system in the early postnatal period [49], while gangliosides play an important role in ongoing neuroplasticity and axon–myelin adhesion and wrapping [47,48,50,51]. Supplementation of gangliosides and phospholipids has been, in fact, shown to produce significant improvement in cognitive processes such as learning and memory and brain growth in preclinical models [52,53,54,55]. In addition, the evidence for calcium, phosphorus, and beta carotene, and their role in brain development is emerging. β-carotene has, so far, been shown to have a weak association with myelin across ages. It is a dietary carotenoid routinely found in human milk, and, together with lycopene, accounts for 70% of the total carotenoids in colostrum [56]. Its role in brain development has only been reported recently, in primates. β-carotene can improve the development of cerebral functional cortical networks assessed by using functional MRI (fMRI) in formula-fed infant macaques [57]. Brain levels of β-carotene were positively correlated with connectivity in the auditory–limbic network and the insular-opercular and dorsal-attention networks, key networks involved in social–emotional skill development [57]. Furthermore, recent clinical and preclinical evidence suggests that β-carotene supplementation may improve social and emotional skills in autism [58,59]. Lastly, 3′-Sialyllactose (3′-SL) and 6′-Sialyllactose (6′-SL) have been reported to be crucial for brain and cognitive development. 6′-SL has been shown to impact myelination in a preclinical study, potentially via a reduction in sialylated binding targets for the myelin-associated glycoprotein [60], while 3′-SL has been found to impact positively cognitive development in infants [61].

Notably, the findings of this study reinforce the previous notions on how nutrition influences brain development and cognitive functions. However, it provides, in addition, evidence of the sensitive periods during development in early life providing the link between specific nutrients intake and social brain regions. This innovative approach is fundamental for advancing effective nutritional interventions and reliable future brain nutrient recommendations.

### 4.1. The Dynamic Pattern of Myelin–Nutrient Intake Associations: Three Age Windows

Besides the 16 continuously important nutrients across ages 6 to 60 months, most assessed nutrients followed a dynamic association pattern with MWF. Of note, three nutrient–myelin age windows were identified with 28 nutrients associated with myelin across the ages from 6 to 20 months, dropping to 14 nutrients from 21 to 30 months, and doubling to 27 nutrients from 31 to 60 months. All classes of nutrients showed dynamic patterns across these three sensitive age periods—except for 3′-SL, which was significantly associated with myelin in every window. For children aged 6 to 20 months (window 1), nutrients relevant for social brain development mainly included micronutrients (vitamins and minerals) followed by amino acids and 3′-SL. No lipids were associated with myelin in the social brain areas at this very early life stage. Micronutrients are well known to be crucial for brain development, including window 1 nutrients such as iron [62,63], zinc [64], folate [65], vitamin B1 [66], vitamin B2 [67], vitamin B6 [68] and copper [69], as their deficiencies can lead to different neurodevelopmental disorders or deficits. These micronutrients are also implicated in the development of social–emotional skills. However, most evidence comes from children with neurodevelopmental disorders or malnourished children. Overall, the emerging evidence shows that micronutrients such as iron, zinc, folate, vitamin B1, vitamin B2 and vitamin B6, either individually or in a blend consumed by older children, could influence social–emotional development and aid social–emotional skills in neurodevelopmental disorders [70,71,72].

Prospective studies, including dietary micronutrient interventions in healthy children, are required to make definite conclusions. For essential amino acids of window 1 (histidine, leucine and isoleucine), to our knowledge, there are no studies assessing their effects on social–emotional skills in children. However, studies in adults show that these essential amino acids might have a beneficial effect on both cognition and social–emotional functions. For example, a double-blind, randomized, placebo-controlled trial showed that oral administration of seven essential amino acids, namely, leucine, phenylalanine, and lysine, isoleucine, histidine, valine, and tryptophan, improved positive emotions (World Health Organization-Five Well-Being Index) and social interactions in adults [73]. Furthermore, both leucine and isoleucine are significantly decreased in patients with major depression in comparison with healthy subjects, and studies show a significant negative correlation between their concentrations and depression scores [74]. The effects of leucine supplementation have been also studied in subjects with cerebral palsy in relation to well-being, and showed a positive effect on fatigue, sleep quality, stress levels and mood [75].

Several models can be considered to understand how essential amino acid supplementation improves psychosocial function. The intake of essential amino acids can directly affect brain function through mRNA translation, support for myelination, neurotransmitter synthesis, and competitive inhibition of neurotoxic substance influx into the brain (such as kynurenine, that shares the same transporters with some amino acids) [76]. However, the exact mechanisms underlying this relationship are unclear. What is known is that histidine is a precursor of histamine, which functions as a neurotransmitter in the brain and is involved in the formation of myelin and multiple brain functions [77,78]. Similarly, histidine is also implicated in depression [79]. Histidine residues are important for the maintenance of the myelin sheath, as they participate in the hydroxylation of the galactosylceramide, which is responsible for the compaction of the myelin [80]. Likewise, branched-chain keto acid dehydrogenase kinase (BDK) deficiency has been shown to reduce branched-chain amino acids (including isoleucine and leucine) and to cause myelin deficiency and diminished expression of myelin basic protein in mice [81].

There is a scarcity of studies investigating the effects of lipids on social–emotional development, with some positive evidence reported for DHA [82], sphingomyelin [23,83] and phosphatidylserine [84]. Notably, for children aged 21 to 30 months (age window 2), lipids become associated with myelination, while vitamins, minerals and amino acids become non-significant. For older children aged 31 to 60 months (age window 3), the nutritional effects seem like the previous age stage but more diversified—with lipids continuing to be necessary, followed by a contribution and re-appearance of amino acids and a small number of micronutrients. Among the body organs, the brain is one of the richest in lipids [85]. The myelin sheath formation requires high levels of fatty acid and lipid synthesis as well as the uptake of extracellular fatty acids [86]. Preclinical studies suggest that a diet deficient in essential fatty acids during development can cause hypomyelination and dietary fatty acids to be important in central nervous system myelinogenesis [87]. Hence, it is not surprising that lipids take a key role in myelin development in the social brain regions after the first 20 months of age, when the development of social–emotional skills is accelerated and reaches its peak window of brain sensitivity. Our results further support the key role that lipids play not only during infancy, but also for toddlers.

To understand the level of importance of nutrients associated with myelin, we performed the nutrient blend identification analysis. This allowed us to reduce the associated nutrients to the minimum core group necessary to affect myelin positively. We could highlight which groups of nutrients and nutrient categories have the most substantial effect on the myelin development of the social brain regions, using targeted and non-targeted approaches (Table 3 and Table 4). This knowledge can be substantiated by future nutritional interventions or research studies exploring brain–nutrient dynamics.

Understanding sensitive periods for the specific link between nutrients and social brain maturation is key for developing effective nutritional solutions that may support social and emotional development. The dynamic relationship between nutrition and social and emotional skills has been previously described in malnourished children [88] and children with neurodevelopmental disorders [89], but not in healthy and well-nourished populations. Furthermore, although the notion of the dynamic interplay between nutrition, brain and skill development has been hypothesized and recently shown for the whole brain myelin [21,24], this is the first study to support the age and social brain-stage-appropriate nutrition that can be relevant for social–emotional development. This study proposes the dynamic nutritional needs of the young developing brain when the timing is crucial and brain development active, as highlighted by nutritional deficiencies and intervention studies [27,90,91].

### 4.2. Social Brain in Neurodevelopmental Disorders and the Role of Nutrition

The social brain is a set of interconnected neural regions and circuits necessary for processing social information [92]. It consists of a collection of distributed structures, circuits, and networks [93]. The regions of the social brain include the amygdala, superior temporal sulcus, superior temporal gyrus, fusiform face area, and temporal pole [93], as well as the anterior cingulate cortex, insula, and medial prefrontal cortex [92,93]. These brain regions are involved in processing social information, such as facial expressions, voice, and body language [92,94]. Recent research has identified the right orbitofrontal cortex as a brain region associated with social regulation and social cognition [93]. Prematurity has also been associated with altered brain volumes in many regions associated with the social brain, suggesting that the social brain circuits may have developmental susceptibilities to the insults associated with preterm birth [93]. Furthermore, social behavior deficits are a salient feature of a range of neurodevelopmental and psychiatric disorders such as autism spectrum disorders (ASD), schizophrenia (SCZ) and attention deficit hyperactivity disorder (ADHD), among others [93]. Therefore, the social brain is significantly affected in neurodevelopmental disorders such as ASD. The “social brain” hypothesis proposes that the demands of the social environment provided the evolutionary impetus for the emergence of the complex circuitry of a “social brain” [95]. This is evidenced by the fact that neurodevelopmental disorders such as autism and schizophrenia are characterized by abnormal social cognition and the corresponding deficits in social behavior [96]. This has led to the development of a focus on disorders that impact social behavior [97,98,99]. It is hypothesized that these forms of deviation from normal social brain development in either direction are mediated in part by genetic and environmental factors. However, the exact mechanisms that contribute to these deficits are still not clear.

Over the past decade, imaging studies have shown that functional connectivity and microstructural deficits in white matter are hallmarks of patients with neurodevelopmental disorders [99,100,101]. Of note, deficits in social behavior and cognitive performance have been associated with reduced myelination and impaired connectivity in patients with ASD and SCZ, but also in preterm infants that show a higher prevalence for ASD [93]. Recent studies suggest that autoantibodies to myelin basic proteins (MBP) are significantly more present in autistic children than in control subjects [102], suggesting that MBP is a potential candidate autoantigen in autism [102,103]. Furthermore, it has been suggested that oligodendrogenesis and myelination significantly contribute to the observed outcomes in autism [99,103]. Early cerebellar injury or dysfunction are other factors associated with a high relative risk for autism, surpassed only by genetic factors [103]. In addition, transient white matter hyperplasia, myelin thickening and/or over-expression of oligodendrocyte lineage and myelin markers have also been documented in the cerebellum of patients with ASD and several genetic ASD-like mouse models [103].

The role of nutrition in social behavior and neurodevelopmental disorders is a new, developing research field [104]. Studies demonstrate that breastfeeding experience is associated with decreased risk of atypical social development and ASD [104]. Specifically, limited or lack of consumption of colostrum or first milk by newborns may increase the likelihood of developing ASD, with evidence suggesting that the absence, or short duration, of exclusive breastfeeding may be associated with the development of ASD [104]. Research has also shown that breastfeeding duration significantly reduces the likelihood of developing ASD, and that children with over 6 months of exclusive breastfeeding or formula supplemented with DHA exhibit the lowest probability for subsequently being diagnosed with ASD [14]. Additionally, nutritional programming in early life may influence cognitive function and predispose genetically susceptible individuals to ASD [105]. Polyunsaturated fatty acid (PUFA) deficiencies during pregnancy can lead to reduced cognitive functioning and learning and memory capabilities, if not corrected in early development [105]. Additionally, maternal obesity is related to the development of ASD due to fetal brain inflammation [105]. Preliminary data suggest that omega-3 supplementation may improve hyperactivity, lethargy, and stereotypy in children with ASD [105]. However, the role of nutrition in social behavior and autism is still inconclusive, as studies exploring the effect of nutritional therapies on differing aspects of ASD rarely compare effectiveness with that of other interventions [103,104,105].

Our findings provide a better understanding of the effect of nutrition on myelination that involves positive nutrient correlations during the nutrient–myelin windows [21] and could be beneficial also for individuals with ASD, except for the healthy population.

### 4.3. Limitations

We recognize that our study has limitations that it is important to take into consideration to ensure more reliable conclusions about the relationship between nutrition and brain development. Limitations of our work include the need for longitudinal and behavioral social–emotional data and data limited to the US population. Nutritional intake varies across geographies; therefore, generalization of the findings to other populations is limited to when intake values are comparable. In addition, another limitation is related to dietary data collection, e.g., 24 h recall may not best capture a typical day. Self-reported dietary intake can often be inaccurate due to factors such as memory bias, social desirability bias, and difficulty in estimating portion sizes. Parents may also have difficulty accurately reporting the type and amount of foods their child consumes, especially if the child eats meals outside of the home or with other caregivers. Therefore, future research could consider incorporating additional measures of nutrient intake, such as biomarkers or nutrient-specific assessments, and validating the self-reported data with other methods. In addition, having a limited data collection timeframe of “within 1 week of the neuroimaging data” might not be sufficient to capture a comprehensive and representative picture of the participants’ usual dietary habits, as nutrient intake can vary significantly from day to day. Future research in this area should consider extending the data collection timeframe to better capture the variability in dietary habits.

Future studies should replicate the findings using multimodal approaches not limited to myelination as a marker of social brain maturation and include the behavioral assessment of social–emotional skills.

## 5. Conclusions

Nutrient–brain development associations are dynamic throughout child development, suggesting the existence of age and brain stage-adapted nutritional requirements, yet data on healthy young children are scarce. The approach described here provides new hypotheses for nutrients and nutrient combinations potentially relevant for social brain development in early life and may lay the groundwork for future intervention studies and for the further identification of specific patterns of other developmental domains and the interplay with nutritional factors. Furthermore, these findings may help expand nutritional recommendations, currently based on overall health and growth, to brain development specific needs in early life. Validation studies for age-adapted social brain nutrients and their efficacy for social–emotional development outcomes are desired.

## Figures and Tables

**Table 1:** Demographic information for the study population ^a^.

Biological Sex (N)	Male	165
	Female	128
Age (months) (Mean (SD))		25.5 (4.5)
Birth Weight (lbs/kg)		7.4 (1.2)/3.36 (0.54)
Birth Length (inches/cm)		20.2 (1.9)/51.3 (4.8)
Maternal Education (N)	Professional Degree	105
	College Graduate	74
	Partial College	49
	High School Graduate	21
	Partial High School	3
	Not Reported	41
Race (N)	White	181
	Black or African American	36
	Asian	13
	Mixed	22
	Not Reported	41
Participant Cognitive Development Composite (Mean (SD))	MSEL ELC	101 (18)
	MSEL VDQ	99 (22)
	MSEL NVDQ	105 (18)

^a^ Abbreviations: N: number of individuals, SD: standard deviation, MSEL ELC: Mullen Scales of Early Learning-Early Learning Composite, MSEL VDQ: Mullen Scales of Early Learning Verbal Developmental Quotient, MSEL NVDQ: Mullen Scales of Early Learning Nonverbal Developmental Quotient.

**Table 2:** Daily nutrient intake means, and minimum (min)-to-maximum (max) ranges for nutrients per age window ^a^.

Nutrient	Mean (SD)	Min–Max
**Age Window 6–20 Months**		
Copper (mg)	0.6 (0.2)	0.2–1.1
Folate (mcg)	179.9 (85.3)	29.3–459.1
Histidine (g)	0.2 (0.1)	0–0.7
Iron (mg)	9 (5.2)	0.6–23
Isoleucine (g)	0.4 (0.3)	0–1.2
Leucine(g)	0.8 (0.6)	0–3.1
Magnesium (mg)	144.6 (54.8)	33.2–295
Phosphorus (mg)	688 (235.1)	163.8–1349.4
Potassium (mg)	1281.2 (548.7)	131–3268.1
Selenium (mcg)	55.1 (25.9)	6.2–150
Valine (g)	0.5 (0.4)	0–1.5
Vitamin A (mcg)	453.3 (344.5)	30.6–2597.6
Vitamin B1 (mcg)	0.8 (0.4)	0–1.7
Vitamin B2 (mcg)	1.3 (0.5)	0.3–2.5
Vitamin B6 (mcg)	1 (0.6)	0.2–4
3′SL (mg)	15.3 (17.3)	0–54.6
Zinc (mg)	6.2 (2.8)	0.2–13
**Age Window 21–30 Months**		
alpha_lactalbumin (g)	0.3 (0.2)	0–0.9
Cryptoxanthin (mg)	117.3 (102.6)	0.4–387
Gangliosides (mg)	3.1 (2.6)	0.5–12
Oligofructose	1.6 (1.5)	0–5.2
PFA 18:4 (Octadecatetraenoic acid) (mg)	4 (5.8)	0–26
PFA 20:4 (Eicosatetraenoic acid) (g)	0.1 (0.1)	0–0.3
PFA 22:5 (Docosapentaenoic acid) (mg)	4.1 (6.3)	0–42
Phosphatidylcholine (g)	0.5 (0.7)	0–3.5
Phosphatidylinositol (mg)	9.8 (11.8)	0–47.5
Sphingomyelin (mg)	7 (8.4)	0–43.9
3′SL (mg)	20.6 (20.8)	0–90.5
6′SL (mg)	4 (4)	0.1–18.3
**Age Window 31–60 Months**		
Alpha-carotene (mcg)	276.7 (264.1)	0–1669.5
alpha_lactalbumin (g)	0.2 (0.2)	0–0.8
Alpha tocopherol (mg)	6 (2.8)	0–17.9
Gangliosides (mg)	3.1 (2.5)	0.1–13
Histidine (mg)	0.3 (0.2)	0–0.9
Isoleucine (mg)	0.5 (0.2)	0–1.7
Leucine (mg)	1.1 (0.7)	0–2.9
Lysine (mg)	0.8 (0.4)	0–2.3
MFA 20:1 (Eicosenoic acid) (g)	0.1 (0.1)	0–0.5
PFA 18:4 (Octadecatetraenoic acid) (mg)	6.8 (10.1)	0–56
PFA 20:5 (Eicosapentaenoic acid) (mg)	8.3 (15.4)	0–127
PFA 22:5 (Docosapentaenoic acid) (mg)	10.1 (8.6)	0–37
PFA 22:6 (Docosahexaenoic acid) (mg)	32.1 (46.2)	0–255
Phosphatidylcholine (g)	0.6 (0.6)	0–3.2
Phosphatidylethanolamine (g)	0.1 (0.1)	0–0.7
Phosphorus (mg)	894.2 (253.4)	32.5–1473.6
Phosphatidylinositol (mg)	16.5 (22.1)	0–168.3
Phosphatidylserine (mg)	18.3 (34.7)	0–293.4
Sphingomyelin (mg)	9.7 (11.4)	0.1–76.6
Tryptophan (g)	0.1 (0.1)	0–0.6
Valine (g)	0.7 (0.3)	0–2
3′SL (mg)	11.7 (15)	0–81.6
6′SL (mg)	2.7 (2.9)	0–16.5

^a^ Abbreviations: SD: standard deviation, g: grams, mg: milligrams, mcg: micrograms.

**Table 3:** Nutrient combinations from untargeted analyses ^a^.

Nutrient Combinations	Age Window 6–20 Months	Across Age Range 6–60 Months
	Vitamins and Amino Acids	Lipids + HMO
Nutrient	Vitamin B1	Gangliosides
	Vitamin B2	Sphingomyelin
	Vitamin B6	Vitamin B12
	Zinc	Phosphatidylinositol
	Iron	Phosphorus
	Copper	3′SL
	Histidine	
	Isoleucine	
	Lysine	
	Leucine	
Correlation	0.54	0.67
(95% CI)	(0.17, 0.77)	(0.52, 0.78)
RMSE	0.55	0.73

^a^ Abbreviations: HMO: Human Milk Oligosaccharides (HMOs), CI: confidence interval, RMSE: Root-mean-square deviation, 3′SL: 3′-Sialyllactose.

**Table 4:** Nutrient combinations from targeted analyses for main nutrient categories across age windows ^a^.

Nutrient Combinations	Micronutrients	Fatty Acids	Lipids	Amino Acids
	(Vitamins and Minerals)			
Nutrient	Vitamin B12	Linolelaidic acid (18:1)	Gangliosides	Isoleucine
	Phosphorus	γ-Linolenic acid (18:3), Docosahexaenoic acid [DHA]	Sphingomyelin	Tryptophan
	Folate	Arachidonic acid	Phosphatidylinositol	Histidine
	Calcium	Octadecenoic acid (18:1)	Phosphatidylserine	Lysine
	Vitamin A	Gondoic acid (20:1)	Phosphatidylcholine	Glycine
		Palmitoleic acid (16:1)		Leucine
		Docosapentanoic acid (22:5)		
		Eicosapentaenoic acid (20:5)		
		Erucic acid (22:1)		
		Stearidonic acid (18:4)		
Correlation	0.90	0.56	0.57	0.50
(95% CI)	(0.51, 0.78)	(0.38, 0.70)	(0.38, 0.70)	(0.30, 0.66)
RMSE	0.77	0.80	0.76	0.86

^a^ Abbreviations: CI: confidence interval, RMSE: Root-mean-square deviation, DHA: Docosahexaenoic acid.

## Data Availability

The materials necessary to attempt to replicate the findings presented here are not publicly accessible.

## Supplementary Materials

The following supporting information can be downloaded at: https://www.mdpi.com/article/10.3390/nu15173754/s1, Table S1: Inclusion criteria of participants in the study.
