# Supplementary material for: Dynamic Interplay between Social Brain Development and Nutrient Intake in Young Children
Source: Nutrients. 2023 Aug 28;15(17):3754. doi: 10.3390/nu15173754 (PMC10490067; doi:10.3390/nu15173754)
Supplement: Supplementary file 1 [file nutrients-15-03754-s001.zip › nutrients-2527317-supplementary.pdf]

nutrients MDPI

Supplementary Table S1. Inclusion criteria of participants in the study [a].

	Description of inclusion criteria	Condition for inclusion
**1**	Full-term singleton pregnancy	>37 weeks
2	Uncomplicated pregnancy	i.e., no pre-eclampsia, high pregnancy blood pressure or gestational diabetes, GDM report
3	Abnormal fetal ultrasound report	No
4	5-minute APGAR score for infants	≥8 points
5	Birth weight	>1500 grams
6	History of neurotrauma	No
7	Sibling or parental psychiatric history reports, including autism	No
8	Mother with Major depressive disorder requiring medication during pregnancy or in the 6 months prior to becoming pregnant.	No
9	Successful MRI scan with time-matched nutrition intake information using the ASA-24 for at least one 24-hour period.	Performed within one week of the MRI

[a] Abbreviations: GDM: Gestational diabetes mellitus; APGAR: Activity, Pulse, Grimace, Appearance, Respiration; MRI: Magnetic Resonance Imaging
